# Towards application of CRISPR-Cas12a in the design of modern viral DNA detection tools (Review)

**DOI:** 10.1186/s12951-022-01246-7

**Published:** 2022-01-21

**Authors:** Julija Dronina, Urte Samukaite-Bubniene, Arunas Ramanavicius

**Affiliations:** 1 Laboratory of Nanotechnology, Department of Functional Materials and Electronics, Center for Physical Sciences and Technology, Sauletekio av. 3, Vilnius, Lithuania; 2 Department of Physical Chemistry, Faculty of Chemistry and Geoscience, Vilnius University, Naugarduko str. 24, 03225 Vilnius, Lithuania

**Keywords:** COVID-19, CRISPR-Cas, DNA-biosensors, SARS-CoV-2 virus, CRISPR–Cas12a, Bioanalytical system

## Abstract

Early detection of viral pathogens by DNA-sensors in clinical samples, contaminated foods, soil or water can dramatically improve clinical outcomes and reduce the socioeconomic impact of diseases such as COVID-19. Clustered regularly interspaced short palindromic repeat (CRISPR) and its associated protein Cas12a (previously known as CRISPR-Cpf1) technology is an innovative new-generation genomic engineering tool, also known as ‘genetic scissors’, that has demonstrated the accuracy and has recently been effectively applied as appropriate (E-CRISPR) DNA-sensor to detect the nucleic acid of interest. The CRISPR-Cas12a from *Prevotella and Francisella 1* are guided by a short CRISPR RNA (gRNA). The unique simultaneous *cis-* and *trans-* DNA cleavage after target sequence recognition at the PAM site, sticky-end (5–7 bp) employment, and ssDNA/dsDNA hybrid cleavage strategies to manipulate the attractive nature of CRISPR–Cas12a are reviewed. DNA-sensors based on the CRISPR-Cas12a technology for rapid, robust, sensitive, inexpensive, and selective detection of virus DNA without additional sample purification, amplification, fluorescent-agent- and/or quencher-labeling are relevant and becoming increasingly important in industrial and medical applications. In addition, CRISPR-Cas12a system shows great potential in the field of E-CRISPR-based bioassay research technologies. Therefore, we are highlighting insights in this research direction.

**Graphical Abstract:**

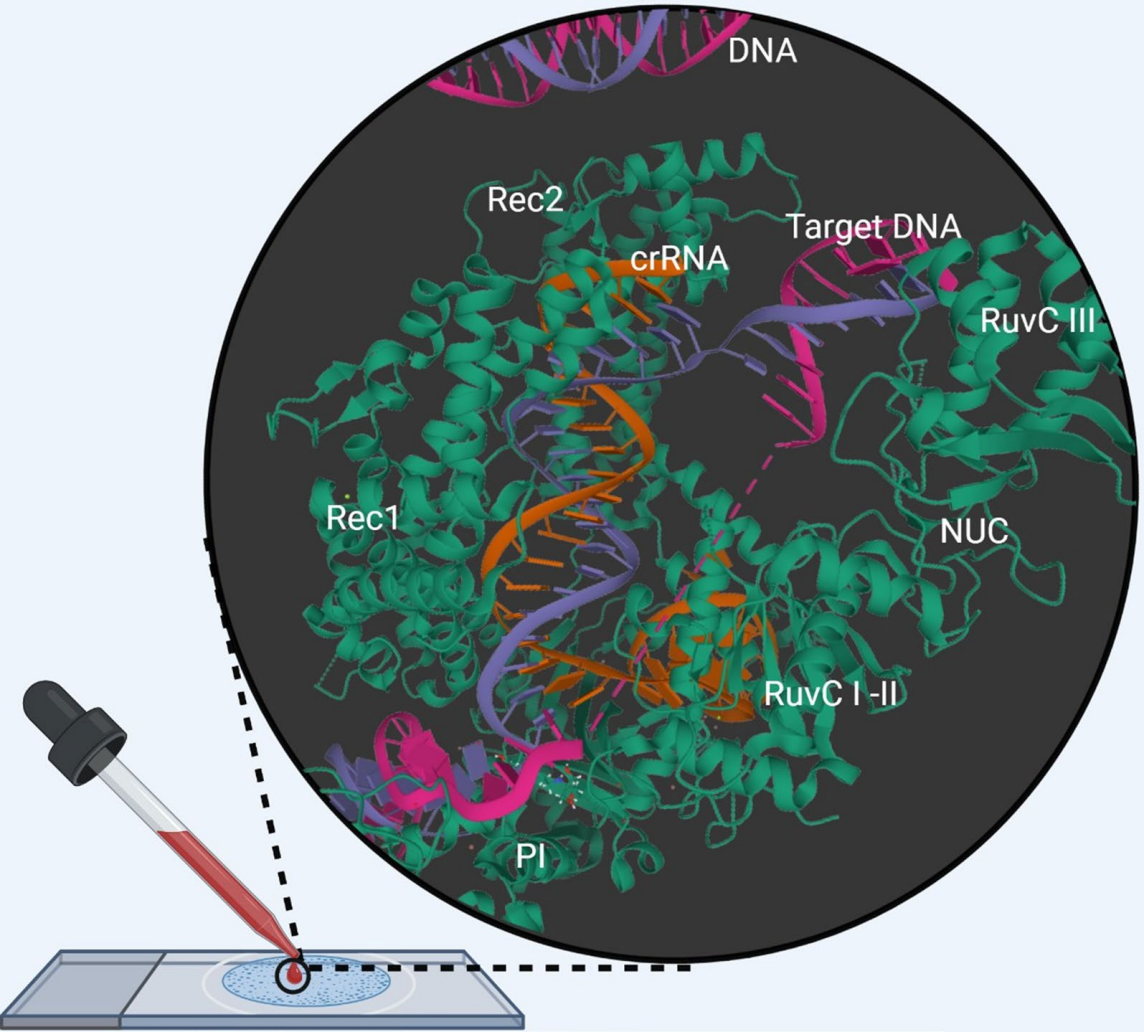

## Introduction

It is estimated that there are ten nonillions (10^31^) individual viruses on our planet, and more than 7000 viral genotypes have been extensively studied and described [1–4]. A virus is an infectious pathogen agent of a non-cellular structure that cannot reproduce outside the host cell. Replication of the viral genome occurs only inside the living (host) cells. They are infectious agents that can infect any cellular organism (prokaryotic, eukaryotic, and archaea) [5–9]. Furthermore, viruses are found in almost every ecosystem on the planet. However, viruses are smaller than bacteria, making them impossible to see under a light microscope, using only electron microscopes (cryo-electron microscopes, transmission electron microscopes) or X-rays to visualize them [10]. Moreover, the origin of the viruses has not been elucidated because they do not form fossils [11], but molecular technology has been most helpful in exploring their origin and creating a classification. However, since 1892 [12], the classification of viruses has changed several times. André Lwoff, Robert Horne, and Paul Tournier [13] were the first who develop a virus classification tool based on the Linnaean hierarchical system. In 1966 when the International Committee on Viral Taxonomy (ICTV) was established, the Baltimore [14] classification system began to be used as a traditional hierarchy of viruses. In most cases, viruses can be grouped according to their genetic material: DNA or RNA.

DNA viruses (herpes viruses, smallpox viruses, adenoviruses, human papillomaviruses, pararetro viruses, Etc.) are responsible for the most significant viral infections. The genome of DNA viruses is based on deoxyribonucleic acid (DNA), and their replication, transcription, and immunization involve DNA-modifying enzymes (DNA polymerases, Reverse Transcriptase, CRISPR-Cas, Etc.) [15], initially can be present in the virus hosting cell and/or possessed by a virus [16–18]. Clustered Regularly Interspaced Short Palindromic Repeats (CRISPR) together with a CRISPR associated protein (Cas) and guided by short CRISPR RNA (gRNA) acts as an ‘immune’ and/or antiviral system of prokaryotic organisms such as bacteria and archaea [19]. However, DNA viruses are very often infecting both prokaryotic and eukaryotic microorganisms and, therefore, the genome of DNA viruses is diverse [18].

The genomes of DNA viruses, which can be single-stranded (ssDNA) or double-stranded (dsDNA), encode only a few genes (proteins) [20–23]. An infectious particle of a virus called a virion consists of a nucleic acid surrounded by a proactive layer of a capsid protein. Capsid (diameter of 20 to 300 nm) [24] is the protein envelope of the virus that encloses its genetic material. It consists of several oligomeric (repeating) structural units made from proteins called protomers. Protomers are made up of identical protein subunits called capsomeres. Viral genomes are circular, as in polar viruses, linear, and adenoviruses [25–27]. Most viruses control cellular mechanisms for macromolecular synthesis in the late phase of infection, directing it to synthesize large amounts of viral mRNAs and proteins rather than thousands of normal cellular macromolecules. Viruses often express proteins that modify host cell processes to maximize viral replication [28–31]. In many cases, the replication of the viral genome of most DNA viruses takes place in the cell nucleus, and here, viruses are completely dependent on host cell DNA synthesis processes. In other cases, DNA viruses from larger genomes can encode most of this cell mechanism by themselves [32]. In eukaryotes, the viral genome must pass through the cell nucleus membrane to reach these metabolic processes, and in bacteria, it must only enter the cell [33–36]. However, viruses use vital metabolic pathways in host cells for replication, making them challenging to eliminate from living organisms without drugs that typically cause toxicity to host cells. The most effective medical approaches to viral diseases are immune-to-infectious vaccines and antiviral drugs that selectively interfere with viral replication. Therefore, early detection of viral pathogens by sensitive bioassay methods in clinical samples, contaminated foods, soil, or water can significantly improve clinical outcomes and reduce the socioeconomic impact of viral diseases.

Quantitative real-time PCR (qPCR) [37], next-generation sequencing (NGS) [38, 39], enzyme-linked immunosorbent assays (ELISA) [40] are currently the most widely used “gold standard” methods, which are applied for the detection and identification of viral DNA in clinical practice [41–43]. Therefore, during the recent epidemic of COVID-19 [44, 45], it is especially relevant to develop a new method or to adapt/optimize other specific, sensitive, rapid, inexpensive, accurate, already applicable techniques for early detection of a nucleic acid of interest in specific and environmentally friendly methods.

Many of the applicable properties listed above are suitable for biosensors based on enzymatic reactions and electrochemical signal determination methods. Electrochemical response-based biosensing platforms are widely used due to their fast performance, affordable system, relatively simple sensing procedures, and direct determination of analytes [46]. One of the critical challenges for such a system is accuracy. However, the applicability of various enzymes combined with inorganic (silica [47], gold [48–50], carbon [51, 52]) nanoparticles for biosensor design has been successfully evaluated in many types of research [51, 53–57]. These studies have shown that biosensors combined with enzymes and gold-nanomaterials increase the bioassay system's accuracy, specificity, sensitivity, and selectivity [58, 59]. Hence, gold-based nanomaterials have demonstrated good performance in many applications and may be an attractive candidate for developing a CRISPR-Cas12a based system for several reasons. Gold nanoparticles are stable material, and it is easier to control particle size and composition by synthesis [60, 61]. The most advantageous property of the gold-based nanomaterial is their biocompatibility with various biomolecules [62]. Therefore, gold-based nanomaterials can be used the further developments of biosensors based on DNA- and RNA-modifying enzymes.

Some reviews on CRISPR-Cas diversity, classification, and evolution have been published over the last three years [63–65]. In 2017, to systematize the classification, all complex encoded effector proteins and representatives have been divided into two classes (class 1 and 2), types I-VI, and more than 30 subtypes. Typical class 1 CRISPR-Cas system members are based on a complex of 4–7 Cas proteins (several Cas proteins and crRNAs bind together and form a functional endonuclease). Members this class 1 are widespread in bacteria (including hyperthermophiles) and archaea. Members of the class 2 CRISPR-Cas system use a single multidomain effector protein (uses a single Cas protein with crRNA) and are widespread only in bacteria. Unlike other classes, candidates of the CRISPR-Cas class 2 system (Cas9, Cas12, Cas13) are the most common and best-studied and are described and named as the best candidates for the development of genome editing tools suitable for applications in vivo and in vitro [66].

This review purposely shows and discusses previous research of CRISPR-Cpf1 (Cas12a) and even provides possible ideas for further development. The study addresses the attractiveness of the CRISPR-Cas12a system for simultaneous *cis-*(target) and *trans-*(non-target) DNA cleavage, sticky-end (5–7 bp) employment for the development of potential versatile electrochemical biosensing platforms (E-CRISPR) as DNA-sensors for the verification of single-stranded and double-stranded DNA virus-induced infections and the discovery of any other DNA-targets.

## Main

### CRISPR-Cas action mechanism

**Fig. 1:**
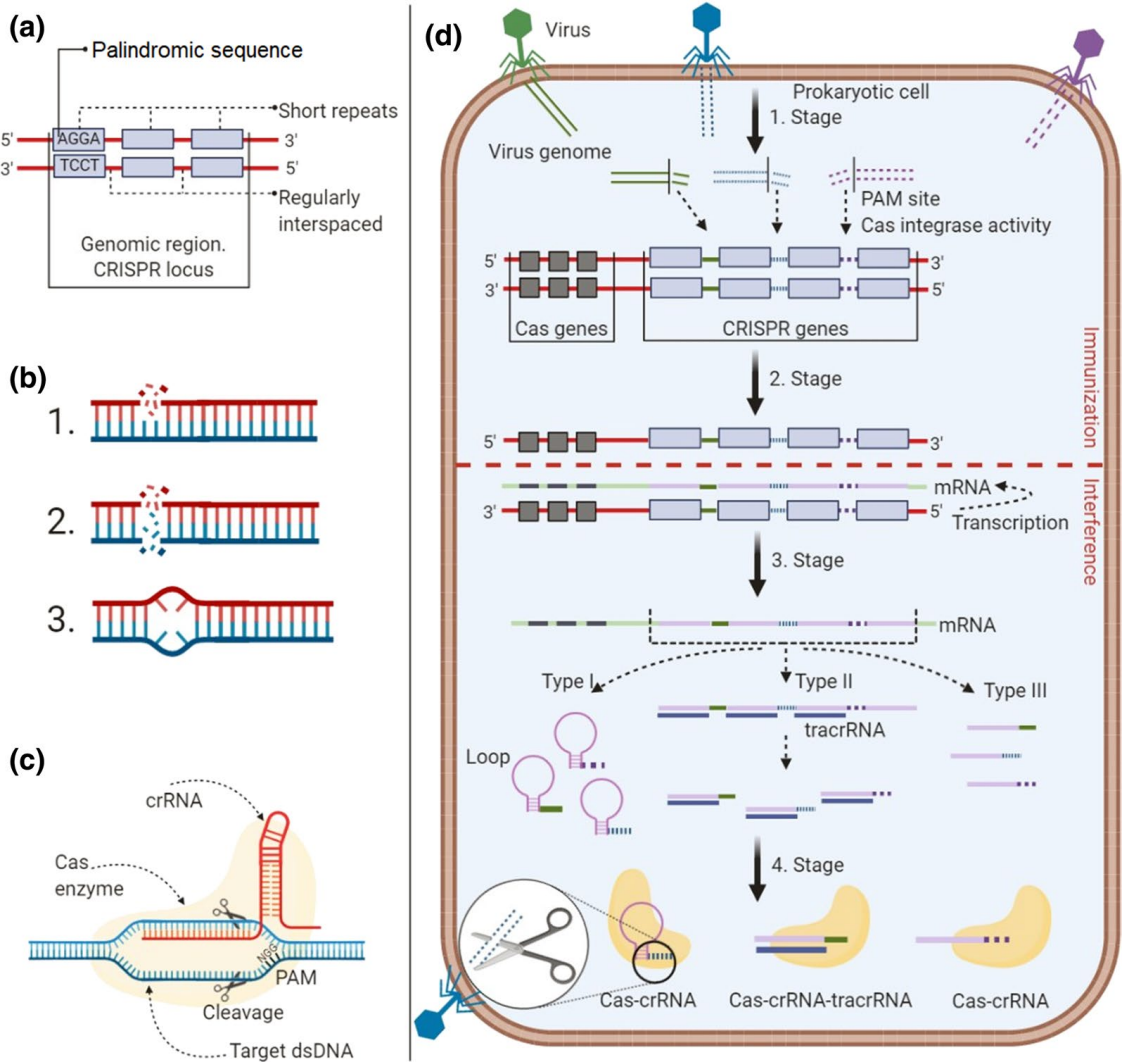
Performance of basic CRISPR-Cas system: **a** Overview of schematic CRISPR definition; **b** Cas proteins are nucleases, endoribonucleases, helicases, or/and integrases: (1) single-strand DNA (break) by nuclease activity; (2) double-strand DNA (break) by nuclease activity; (3) double-strand DNA unwinding by helical activity; **c** Structure of Cas protein, guide RNA (crRNA) and target DNA complex; **d** Performance of four-part CRISPR-Cas action mechanism in the prokaryotic cell: 1. Stage—adaptation; 2–3. Stages—expression; 4. Stage—interference

The CRISPR [67] is a genomic region in the prokaryotic cell where genetic information about adaptive immunity is stored. The system was discovered in *E. coli* in 1987 [68]. More technically, the CRISPR system comprises regularly recurring palindromic sequence inserts in the genomic region (Fig. 1a). The palindromic repeats in the prokaryotic genomic region consist of about 21–40 bp, and regular DNA spacer repeats are about 20–58 bp in length [69]. In the prokaryotic cell CRISPR genomic region, Cas protein is responsible for preserving living cell genetic information [70–72]. In 2007 [73], several in vitro and in vivo studies have shown that the CRISPR-Cas complex acts as an antivirus system in a prokaryotic cell—it detects genetic information from foreign species (e.g., viruses) stores and destroys them in a particular and specific way. Astonishingly, in a prokaryotic cell, the mechanism of action of the antiviral system is efficient and straightforward. When the foreign species attacks the prokaryotic cell, these viruses' genome (RNA, DNA, or plasmids) is injected into the prokaryotic cell. Due to the CRISPR-Cas system, a short piece of foreign species genome information is taken and stored in the memory (locus) of prokaryotic cells, and later it can prevent the cell from repeated attacks by the same strain of viruses. Naturally, in the prokaryotic cell, protection from the foreign genome system is based on three stages of action: adaptation, expression, and interference (Fig. 1d) [74, 75].

### The immunization and interference in prokaryotic cells

The primary mechanism of CRISPR-Cas action was defined by Brouns et al. [76], McGinn et al. [77], Yan et al. [78] and Siksnys et al. [79–82]. The Cas enzyme complex (which always requires Cas1 and Cas2) in the prokaryotic cell is involved in the natural metabolic process during the adaptation stage (Fig. 1d). Incredibly, in the Cas enzyme complex, Cas1 nuclease has integrase activity, and the Cas2 nuclease has endoribonuclease activity, which acts only together. This Cas complex recognizes short motifs (2–4 bp in length) adjacent to the protospacers [83–85]. Cas1 nuclease cleaves a piece of foreign species genome near the PAM and integrates it into the CRISPR locus, yielding a new spacer. In a prokaryotic cell, the CRISPR locus is the area where the peace of genetic information of infecting-foreign species is ‘preserved’ to protect cells from recurrent infections of the same infectious species), a CRISPR RNA is transcribed and processed into mature RNA (crRNA) [86, 87]. During the expression stage (Fig. 1d), a CRISPR RNA (crRNA) molecule is formed. One of the DNA strands with CRISPR locus is transcribed into mRNA (described in some references as pre-crRNA) [77, 88, 89]. mRNA becomes long and exactly complementary to DNA strand, containing repeats of many CRISPR complementary sequences and genomic sequences of foreign species. crRNA is formed from the transcribed mRNA. However, the final composition of crRNA also differs for different Cas types (I–III types). In the expression stage (type I), crRNA consists of one repeat of the CRISPR genome and one genome of a foreign species. Technically, each repetition of the CRISPR sequence forms a loop, but each repetition of the genome sequence of a foreign species does not form a loop, and subsequently, the Cas6e and Cas6f nucleases digest the crRNA. The transactivating CRISPR RNA (tracrRNA) molecule plays a crucial role in the type II process. Technically, the tracrRNA sequence is digested with Cas9 nuclease and RNase III. In the type III process, the Cas6 nuclease directly disrupts each repeat of the CRISPR sequence and the foreign species genome sequence (Fig. 1d) [78, 90]. In the interference stage (Fig. 1d), specially encoded crRNA (as a guide-RNA) is integrated into the Cas protein and forms the CRISPR-Cas complex [91]. In the combined system, the CRISPR-Cas complex contains genomic information from the foreign species recorded into crRNA that allows the foreign genome's identification, detection, and inactivation. This antiviral mechanism works in prokaryotic cells.

Following the present invention, bioengineered CRISPR-Cas systems have been implemented in industries that exploit bacterial cultures (dairy products, agriculture, Etc.) to establish the ability to protect a bacterial culture from virus attack [73, 92–94]. Later, in 2014, the CRISPR-Cas system was adjusted as a powerful tool for genomic research to silence and/or edit gene sequences with additional effectors in various organisms [85, 95–97]. Initial studies in bacterial cell lines [98, 99] and mammalian cells [100–104] have shown that the biologically engineered CRISPR-Cas technology has future potential for correcting gene mutations. Such as malaria blocking genes in mosquitoes [105–110], genome editing in zebrafish [111–113], removing HIV genes [64, 114–116], hepatitis C virus [117] or Parkinson’s disease [118]. However, this exciting progress may have unintended consequences and impacts due to ‘off-target’ effects, which recently are the main limitations of the CRISPR-Cas system because applied genetic corrections can have unpredictable results for future generations. Nevertheless, some developers of the CRISPR-Cas system (Emmanuelle Charpentier and Jennifer A. Doudna) have been awarded by Nobel Prize in Chemistry 2020 [119] and the CRISPR-Cas system has become a new generation of genomic engineering tools.

Furthermore, due to urgent need, chased by pandemics and pathogenic viruses has increased the demand for rapid, accurate, low-cost nucleic acid detection methods, and the studies have shown how the CRISPR-Cas (including Cas12a) combination in DNA Endonuclease-Targeted CRISPR Trans-Reporter (DETECTR) [120], one-Hour Low-cost Multipurpose highly Efficient System (HOLMES) [121] assays can be adapted to become an excellent biomedical diagnostics tool.

### Potentials and limitation of CRISPR-Cas12a

**Fig. 2:**
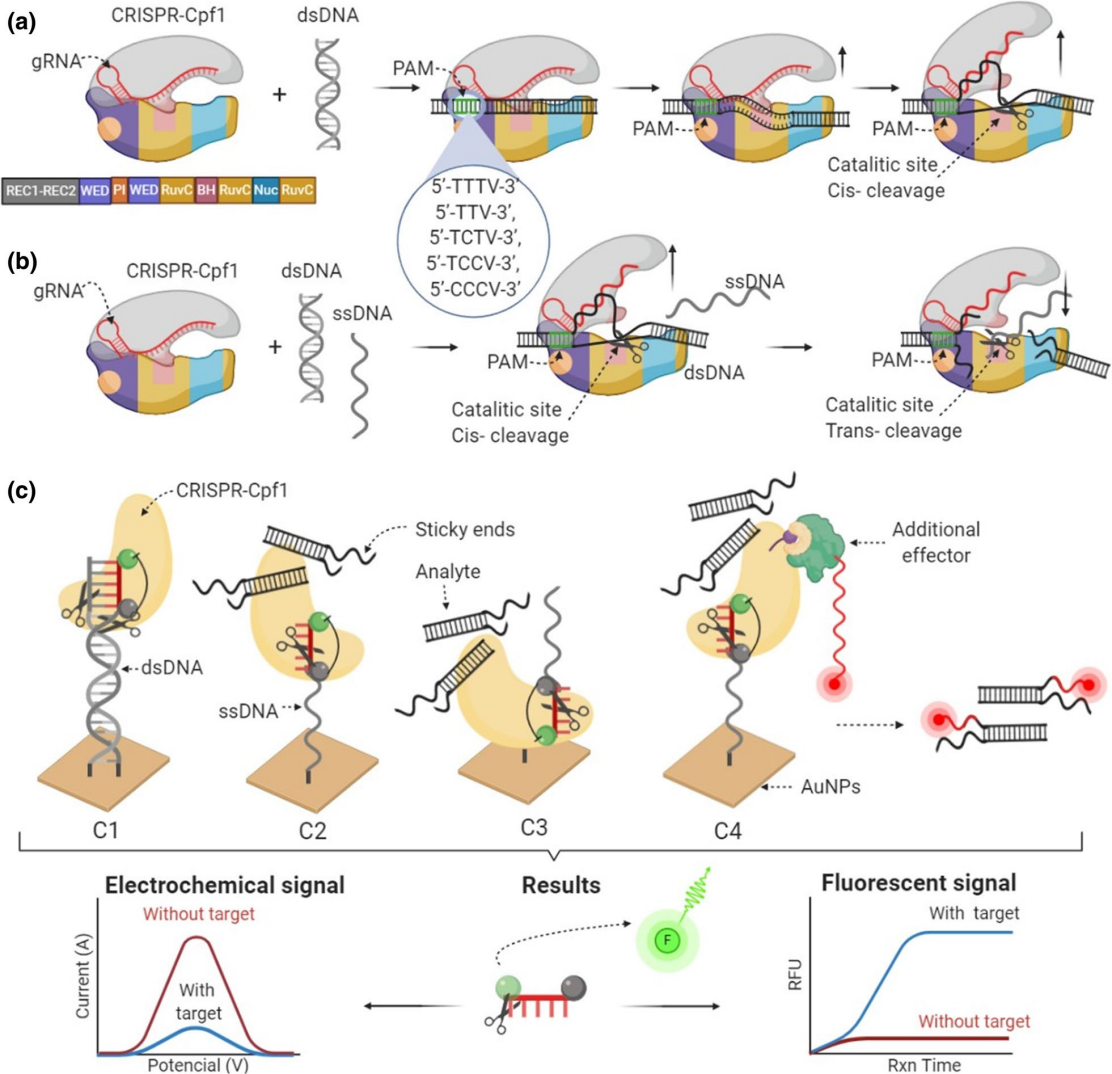
CRISPR-Cas12a resembles the beak structure: the active center is suppressed in the closed position, and the active center is released—in the open position. The **a** N-terminal recognition (REC) region is divided into two [Rec1 (13 α helices) and Rec2 (10 α helices and 2 β strands)] alpha-helical domains that form an antiparallel sheet at the top of the structure. The C-terminal NUC lobe is divided into Wedge [WED (7 α helices and 2 β strands)], PAM-interacting [PI (7 α helices and β hairpin)] and an endonuclease domain involved in DNA repair RuvC (three motifs (RuvC I–III), which form active endonuclease center) and Nuc at the bottom of the structure. Modified bridge helix (BH consist of Arg951 and Arg955 which interact with the phosphate backbone of the target DNA strand) region is in the middle of NUC and REC lobes. The cleavage mechanism: **a** *cis*-cleavage in RuvC domain; **b** *cis*- and *trans-*cleavages in RuvC domain; **c** E-CRISPR biosensor prototype based on CRISPR-Cas12a system: C1—target dsDNA detection by *cis*- cleavage when target dsDNA is immobilized on AuNP as analyte. C2—target dsDNA detection by *trans-*cleavage when ssDNA is immobilized on AuNP and dsDNA is analyte. C3—target dsDNA detection by *trans-*cleavage when CRISPR-Cas12a is immobilized on AuNP and dsDNA is analyte. C4—dsDNA detection by *trans-*cleavage and additional effector when ssDNA is immobilized on AuNP and dsDNA as analyte. The CRISPR-Cas12a system can be fused with some newly designed enzymes like polymerases, other nucleases, or fluorescent proteins as additional effectors. Afterward, modified Cas protein in the CRISPR-Cas system can be used to transport those effectors to a specific DNA sequence for transcription, specific hydrolysis, visualization, or another practical purpose target. Together, through the examples detailed above, we have illustrated integrating CRISPR-Cas systems into different types in vivo biological sensing scenarios as well as emerging monitoring points compassionate and selective diagnostic programs determination of nucleic acids, proteins, and other small molecules

A new potential for the Class 2 CRISPR-Cas system is the Cas12a member (CRISPR from *Prevotella* and *Francisella 1*), which consists of 1300 amino acid residues. This 151 kDa monomeric protein enhances the application of CRISPR systems to genomic engineering [122]. Recently, an opportunity came to follow the crystal structure variants of the *Acidaminococcus sp*. (AsCpf1), which was evaluated by McMahon et al. and Dong et al. [66, 123] and *Lachnospiraceae* bacterium (LbCpf1) evaluated by Safari et al. [124], Jiménez et al. [125] and Swarts et al. [126]. Structural and functional differences in Cas9 and Cas12a were reported [127]. However, the primary key points are that since other class 2 (Cas9, Cas13) candidates Cas12a can be reprogrammed to recognize the target dsDNA sites. A single crRNA guides Cas12a and for cleavage, no tracrRNA is required [128, 129]. Furthermore, Cas12a recognizes the T-rich PAM (5′-TTTV-3′) site by guided crRNA, and another essential distinguishing feature of Cas12a-crRNA is that it can also be directed to suboptimal PAMs (5′-TTV-3′, 5′-TCTV-3′, 5′-TCCV-3′, and 5′-CCCV-3′) sites (Fig. 2a). Moreover, due to these PAM benefits or limitations, Gao and co-authors [130–135] have shown that science can modify robust evolutionary theories and adapt to alternative PAM sequences to increase its targeting range for the CRISPR-Cas12a system, but of course, with a lower cleavage efficiency rate. After the identification of the PAM site, CRISPR-Cas12a cleaves (42–44 bp) target (cis-) double-stranded DNA (dsDNA) of the target at 37 °C temperature and generates sticky ends (5–7 bp) near the PAM target site—this is another essential attribute of Cas12a (Fig. 2a). Hence, the CRISPR-Cas12a is different from other CRISPR-Cas systems identified as additional non-target (trans-) cleavage activity (Fig. 2b), and the protein-based part CRISPR-Cas12a is smaller than that of Cas9. Therefore, the formation of the CRISPR-Cas12a complex with crRNA is remarkably more uncomplicated. The complex formed is smaller, and crRNA forms only one loop. Furthermore, Cas12a has a protospacer (24 bp) and is more specific because Cas12a has a lower intrinsic tolerance for crRNA-target DNA mismatches and requires higher complementarity. The RuvC domain (lysine residue) is responsible for target cleavage, but differently from some other Cas representatives, Cas12a lacks the detectable second endonuclease domains (HNH) [136–138].

CRISPR-Cas12a based quantitative kinetics analysis consists of the following physical and chemical steps: PAM recognition, dsDNA-target binding, R-loop formation (0.1 s^−1^), rejection, cleavage (1.45 min^−1^), and release between crRNA and dsDNA assisting Cas12a already established by Li et al. [54], Swarts et al. [139], Singh et al. [140] and Chen et al. [141].

Direct comparison as mentioned above is unique in the way that the CRISPR-Cas12a system at the RuvC domain has additional nonspecific (*trans-*) single-stranded DNA cleavage activities dependent on Mg^2+^ and Ca^2+^ ions (Fig. 2b) [139, 142, 143]. Recent studies [144–147] have shown that the additional cleavage phenomenon is induced in rapid and complete cleavage of the ssDNA strand when the ssDNA sequence is not complementary to crRNA or other strand sequences. Furthermore, the cleavage doesn’t relate to the dsDNA specific sequence. Technically, no additional PAM sequence is required for self-cleavage activation. After target dsDNA unwinding and cleavage during the ordinary CRISPR-Cas12a action, the RuvC domain becomes accessible, and the non-target ssDNA cleavage occurs spontaneously (Fig. 2b). The spontaneous cleavage indicates that activation of nonspecific ssDNA cleavage has happened in the presence of CRISPR-Cas12a target sequence for dsDNA recognition and cleavage (Fig. 2) [122, 148]. Moreover, in 2020 Christopher W. Smith and co-authors [149] research proved that this CRISPR-Cas12a trans-cleavage is not limited to ssDNA substrates, and Cas12a-based diagnostics can be extended to ssDNA/dsDNA hybrid substrates. Several variables of NaCl (50–150 mM) concentration and fluorescently silent ssDNA/dsDNA (0–12 bp nicked) hybrid substrates were applied in the bioassays to monitor CRISPR-Cas12a *cis-*(target) and *trans-*(non-target) activities. These studies have proved that CRISPR-Cas12a activity significantly reduced the increase in NaCl concentration. This CRISPR-Cas12a *trans-*ssDNA-cleavage activity offers a new strategy to improve transcription and replication responses in vivo, label ssDNA, or develop faster, more sensitive and specific tools for the determination of specific DNA sequences.

In the studies by Gootenberg et al. [150], Kim et al. [151] and Doudna et al. [152], both Cpf1 orthologs (AsCpf1—from *Acidaminococcus sp*. and LbCpf1—from *Lachnospiraceae* bacterium) have been applied in both (i) genome editing in vivo and (ii) DNA assembly in vitro. AsCpf1 and LbCpf1 differences were reviewed in 2007 by Kim et. al. [153] and Verwaal et. al. [154]. However, gene-editing studies in combination with CRISPR-Cas12a have shown the potential for ‘self-processing’. The system can be assembled into a single and relatively simple plasmid suitable for transfection into selected cells to manipulate selected genes. Therefore, the CRISPR molecular tool can detect selected nucleic acid sequences, target gene editing, and a new protein detection strategy. These benefits and unique features increase the attractiveness of the CRISPR-Cas12a system to develop various biotechnological and bioassay research tools [155].

### Analytical applications of CRISPR-Cas12a

CRISPR-Cas systems for nucleic acid detection have been critically discussed by Li et al. [54], Zhang et al. [156], Doudna et al. [141], Gallego et al. [157], Gootenberg et al. [158], Bonini et al. [159], Collins et al. [160], Wang et al. [161] and other authors [145, 162–176]. Here, we review the main powerful, sensitive analytical methods of CRISPR-Cas: (i) fluorescence in situ hybridization (DNA-FISH) assay based CRISPR-Cas9 technique for specific targeting with SYBR green I as a fluorescent probe (detection limit 10 CFU/ml); (ii) CRISPR-Cas triggered isothermal exponential amplification reaction (CAS-EXPAR) based CRISPR-Cas9 technique and isothermal exponential amplification with SYBR green as a fluorescent probe for a large number of DNA generation and target DNA detection at attomole (aM) sensitivity; (iii) CRISPR rolling circular amplification (CRISPR-RCA) assay based CRISPR-Cas9 technique and rolling circle amplification with SYBR green as a fluorescent probe for a large number of DNA generation, amplification; (iii) DNA endonuclease-targeted CRISPR trans-reporter (DETECTR) and one-hour low-cost multipurpose highly efficient system (HOLMES) based CRISPR-Cas12a technique and target nucleic (DNA or RNA) is amplified with isothermal amplification by RPA (recombinase polymerase amplification) or reverse transcription RPA, the target cleavage fluorescence response generated by ssDNA fluorophore-quencher reporter, detection at attomole (aM) sensitivity; (iv) specific high sensitivity enzymatic reporter unlocking (SHERLOCK) system based CRISPR-C2c2 technique for RNA detection by fluorophore-quencher reporter release and emits fluorescence by RPA; (v) SHERLOCKv2—for nucleic acid sequences detection to applied single reaction by using different Cas enzymes mix (C2c2, Cpf1, Csm6) [177–180]. However, the main challenges in these applications mentioned above are additional purification of the sample, expensive labeling of the target with fluorophores, application of other amplification steps with expensive techniques, and data analysis, which requires practice and knowledge.

### E-CRISPR application

Schematic comparison of Cas proteins in their native forms is detailed in publication by Patrick Schindele et al. [181]. The CRISPR-Cas9 system mediates its function through a single effector Cas9 and two small RNAs, crRNA and tracrRNA. After hybridization, the crRNAR- tracrRNA complex binds to the Cas9 nuclease and binds to its recognition site before the PAM sequence. DNA binding is promoted by a 20 nucleotide reference sequence of crRNA. Cas9 nuclease causes a blunt-ended DSB 3 bp before the PAM sequence. The recognition of the crRNA-tracrRNA-target complex is promoted by the REC (recognition) section, the PI (PAM interacting) domain is responsible for recognizing the PAM. The DSB is mediated by the HNH and RuvC nuclease domains, with the HNH domain cleaving the target and the RuvC domain cleaving the non-target chain. The CRISPR-Cas13 system (Cas13a) mediates its function through a single effector Cas13 and a single crRNA. By combining Cas13 and crRNA, the complex binds to its recognition site on the target RNA mediated by the crRNA sequence. The catalytic site is outside the protein, directed to the surrounding solution, hence, the target RNA is cleaved away from the recognition site. The recognition of the crRNA-target complex is promoted by the REC (recognition) section, the cleavage of the target RNA is performed by the HEPN domain. The CRISPR-Cas12a system mediates its function through a single effector Cas12a and crRNA. By combining Cas12a and crRNA, the complex binds to its recognition site downstream of the PAM sequence. DNA binding is promoted by a 23 to 25 nucleotide reference sequence of crRNA. Cas12a nuclease induces a stepwise DSB distal to the PAM sequence. The recognition of the crRNA-target complex is mediated by the REC (recognition) section, the PI (PAM interacting) domain is responsible for identifying the PAM.

New revolutionary research by Lee et al. [182], Zhang et al. [155] and Dai et al. [183] demonstrates a universal and straightforward endonuclease activity monitoring method with the micro-fabricated gold-working electrode-based three-electrode system, where E-CRISPR modifies the gold-working electrode. In 2020, the invented E-CRISPR method is based on CRISPR-Cas12a-mediated interface and ssDNA reporter cleavage. The authors declared that the E-CRISPR system is suitable for detecting key categories (ssDNA, dsDNA) of biomolecules, providing the potential for implementation in the healthcare industry. Moreover, the unique trans- cleavage nuclease activity allows the use of any ssDNA sequence labeled by fluorescence signal reporter, this part of the E-CRISPR system can be easily replaced by another ssDNA with a signal reporter and applied for the determination of selected ssDNA, dsDNA, or ssRNA. Therefore, this aspect ‘to make E-CRISPR-Cas12a system reprogrammable’ is very attractive.

The evolution of the E-CRISPR system has been demonstrated by applying virus DNA (Human papillomavirus, Parvovirus, Dengue viruses) and proteins (Transforming Growth Factor b1 (TGF-b1) protein, Collagen, Aggrecan, and Bovine Serum Albumin) [92, 184–186]. Virus dsDNA and protein conjugated (immobilized) with DNA aptamer electrochemical (E-CRISPR) detection of methylene blue conjugated to gold nanoparticles (MBAuNP) have been successfully developed and performed by electrochemical signal detection. The above research demonstrated the specificity of the E-CRISPR-Cas12a system at sufficient limits of detection. However, the detected LODs depend on the length and structure of the non-target (trans-) ssDNA strand and applied detection method. Recently, higher efficiencies (of 96%, at 30 pM LOD) were achieved when the ssDNA (of 32 bp length) was constructed in the hair-pin structure. The system additionally contained an RNases inhibitor and was performed by EIS method with Fe(CN)_6_^3−/4−^ as a mediator. If compared, the linear structure of ssDNA (32 bp) under the same reaction condition efficiency reaches 56%. Studies confirmed that the linear non-target ssDNA (18–40 bp in length) was immobilized on the carrier. Target DNA detection efficiency was achieved 30% due to electrochemical current outputs a detection method without Rnases inhibitors or crRNA modifiers. However, when comparing other biosensors based on the CRISPR-Cas12a system, the invented E-CRISPR system showed lower sensitivity due to several following issues. The system should first be well prepared for working with RNA, as RNA is highly unstable in ribonucleases in vivo (by tissue) and in vitro (environment) to achieve sensitivity in the electrochemical response. To achieve a higher sensitivity in recording electrochemical response, the system should first be well prepared. The RNA is highly unstable in the presence of ribonucleases in vivo (by tissue) and in vitro (environment), and the chemical modification of crRNA using phosphorothioate (PS), 2′-O-Methyl (2′-O-Me), 2′-Fluoro (2′-F), S-constrained ethyl (cEt) substitutions at the terminal 5′ or 3′ ends, or internal positions [187], or additional components such as RNase inhibitors should be involved into analytical system. As it is essential to eliminate RNases contamination and significantly increase metabolic stability and expression (in vivo), affinity, extend half-live of the system, mediate high levels of gene editing, or effectively determine the limit of detection by determining CRISPR-Cas12a methods. The Wei et al. [188] declare that the detection limit and the dynamic detection range of the E-CRISPR sensor can be further improved. The authors conducted more detailed comparative studies with the Cas12a and Cas9 systems to evaluate the effect of improving E-CRISPR sensor performance. The principle of the E-CRISPR sensor is the target induced conformational change of the surface signaling probe (containing an electrochemical tag), leading to the variation of the electron transfer rate of the electrochemical tag. To better understand the *–trans* cleavage efficiency, the authors investigated the effect of divalent cation (Mg^2+^) concentration in an in vitro degradation solution, as the catalytic domain of RuvC is known to act on nuclease activity based on a bimetallic ion mechanism. Futhermore, enhancement of the detection signal was observed with increasing Cas12a reaction process up to 1 h. The specific and complementarity-dependent enzymatic activity of CRISPR is exploited beyond the detection limit of conventional electrochemical DNA sensors and, most importantly, the detection accuracy [182, 188, 189].

Firstly, gRNA should be designed to be complementary to the target following a correct PAM sequence. Secondly, the Cas protein with crRNA should be constructed appropriately to recognize the PAM sequence in target nucleic acid, and after target (cis-) cleavage, the non-target (trans-) cleavage is activated. Due to the detection method of E-CRISPR based on non-target cleavage, the different duration time of a non-specific target cleavage differs, and the differences were investigated by Dai et al. [155] and Zhang et al. [187]. At the latest publication, a photoactive methylene blue dye, and biotin [182], were used by several authors, but it is hypothesized that some other redox probes (phenothiazines, ferrocenes, porphyrins, Etc.) can be applied to electrochemical [190] signal registration [189, 190].

CRISPR-Cas biosensing systems are suitable for developing CRISPR-Cas12a point-of-care (POC) test devices with performance equivalent to or better than conventional diagnostics practices. Sensitive and rapid detection of nucleic acids with the naked eye is a new direction in analytical diagnostics. For on-site diagnostics, an ssDNA reporter labeled with a quenched green fluorescent molecule cleaved by Cas12a was introduced, and the resulting green fluorescence can be seen with the naked eye or in 485 nm light [193–195]. Point-of-care testing (POCT) is advantageous in terms of its ease of use, greater approachability on the user's friendly, more timely detection, and comparable accuracy and sensitivity, which could reduce the testing load on central hospitals [196, 197].

The CRISPR-Cas system is programmable, modular, and a specific biological tool for genomic or tissue engineering, bioelectronics, and diagnostics [198]. The CRISPR system is an accessible and powerful tool for regulating biological sensing strategies based on a highly selective sensing mechanism as a functional response. Combining Cas protein with a graphene-based field-effect transistor (FET) has been reported femtomole (1.7 fM) sensitivity of designed analytical system towards target sequence. This was achieved within 15 min lasting action of the sensing system because an increasing amount of formed DNA significantly reduces the conductivity of modified FET-gate. In addition, charged phosphate groups involved in DNA structure affect the gate, and therefore current passing through FET is changing [199]. Some other authors reported that CRISPR-Cas as the programmable and modular tool could be integrated into a set of biosensors, as a nucleic acid-based system for a stem-loop enhanced sensitivity and selectivity through degradation activities [204], moreover, such systems can be applied for detecting various targets, including bacteria, viruses, cancer mutations, and others. High CRISPR-Cas potential in biological sensing technologies is constantly inspiring new research activities to develop a new generation of nucleic acid detection platforms. However, the drawback of CRISPR-Cas-based systems is related to the relatively low sensitivity of Cas protein. However, most CRISPR-Cas bio-sensitization methods can directly detect nucleic acid targets, which is beneficial in combination with appropriate DNA-amplification methods. Therefore, the most currently used CRISPR-Cas biosensing systems rely on the target nucleic acid amplification, which improves sensitivity. Therefore, this additional amplification step can create some additional drawbacks and can make the system less robust. In addition, CRISPR biological sensing techniques can only be used for known DNA detection sequences that may limit their application in some specific cases [54]. It should be noted that during the development of ‘hands-on diagnostic systems’, the development of the suitable strategy to immobilize CRISPR-Cas systems on various interfaces is one of the most challenging key issues.

## Conclusions and future perspectives

The evolution of bioassay methods based on E-CRISPR-Cas12a can be unambiguously extended without any amplification or purification-based steps. CRISPR-Cas12a is an attractive tool for detecting non-target (trans-) ssDNA cleavage in electrochemical signal-based systems. In addition, after the cis-cleavage sticky ends (5–7 bp) in dsDNA are formed, this effect can be efficiently exploited to evolve ultrasensitive biosensors for target DNA detection. CRISPR-Cas12a has successfully demonstrated the potential to be applied in susceptible systems suitable for determining exceptional resolution and time efficiency in conjunction with simple visual signal readings and quantitative determination. However, the CRISPR system has additional potential application, which is still not tapped within bio-electroanalytical methods. We also are predicting that in the very near future, DNA-modifying enzymes, including recently very famous CRISPR-Cas9 and CRISPR-Cas12a systems, which are recently finding many applications for various genome-editing related purposes, will find comprehensive application in the design of ‘programmable DNA- and RNA-sensors.

## Data Availability

Not applicable.
